# *Primula pingwuensis* (Primulaceae), a new species in *Primula* sect. *Auganthus* from Sichuan, China

**DOI:** 10.3897/phytokeys.278.204611

**Published:** 2026-08-25

**Authors:** Si-Yu Zhang, Ming Fang, Ying-Feng Hu, Yang Hu, Jiu-Lin Gu, Zhi-Kun Wu, Jian-Wen Shao

**Affiliations:** 1 School of Modern Agriculture and Food Engineering, Chuzhou University, Chuzhou, Anhui 239000, China School of Modern Agriculture and Food Engineering, Chuzhou University Chuzhou China; 2 College of Life Sciences, Anhui Normal University, Wuhu, Anhui 241000, China College of Life Sciences, Anhui Normal University Wuhu China; 3 Chengdu Shanhualanmanshi Gardening Limited Company, Chengdu, Sichuan 610000, China Chengdu Shanhualanmanshi Gardening Limited Company Chengdu China; 4 College of Pharmacy, Guizhou University of Traditional Chinese Medicine, Guiyang, Guizhou 550025, China College of Pharmacy, Guizhou University of Traditional Chinese Medicine Guiyang China

**Keywords:** Phylogeny, *
Primula
*, sect. *Auganthus*, Sichuan, taxonomy

## Abstract

*Primula pingwuensis***sp. nov**., a new species of *Primula* sect. *Auganthus* from Sichuan, China, is described. It resembles *P. sinensis* in having palmately lobed leaves and multi-whorled inflorescences but differs by its multi-branched stems, more densely short-pubescent surfaces, thicker chartaceous leaves, narrower leaf blades, and indistinct translucent nectar guides. A taxonomic key covering all nine known species of *Primula* sect. *Auganthus* is compiled herein to support field morphological discrimination. A chloroplast genome-based phylogeny shows that *P. pingwuensis* is sister to *P. rongrong*, and this clade is sister to *P. fujiangensis*. Incongruence between morphology and molecular phylogeny is observed within the section. Leaf-lobing patterns have undergone multiple independent evolutionary transitions during adaptive radiation in karst habitats, indicating that a few morphological traits cannot be used to reliably infer interspecific relationships. The new species is assessed as Data Deficient (DD) following IUCN criteria, owing to the lack of long-term observations of its threat status and population dynamics. This study provides new data for the taxonomy and evolution of *Primula* sect. *Auganthus*.

## Introduction

The genus *Primula* L. is one of the perennial herbaceous genera with the highest species diversity in Primulaceae, comprising approximately 556 species worldwide (POWO 2026). It is mainly distributed across temperate and alpine regions of the Northern Hemisphere (Hu 1990). The Hengduan Mountains of Southwest China and the mountainous areas around the Sichuan Basin represent the core regions for modern differentiation and endemism of the genus, with more than 300 species recorded in China (Hu 1990; Hu and Kelso 1996). The genus shows extensive floral trait variation (e.g., heterostyly and corolla morphology), frequent hybridization and polyploidization, and has undergone adaptive radiation in heterogeneous alpine and karst habitats (Richards 2002; Ma et al. 2014; Gilmartin 2015; He et al. 2021; Zhang et al. 2023). Thus, *Primula* serves as an ideal model for exploring floristic evolution, species differentiation, and adaptive radiation.

*Primula* sect. *Auganthus* Pax ex Balf. f. is a well-defined small section within the genus, morphologically characterized by densely hairy plants, shallowly to deeply lobed leaves, and calyces with distinctly broad and inflated bases (Balfour 1913; Balfour and Farrer 1918; Hu 1990; Hu and Kelso 1996). Members of this section are mainly confined to karst habitats in the mountainous border areas of Sichuan, Hubei, and Shaanxi provinces (Hao et al. 2002; Gu et al. 2025; Zhang et al. 2025; Sheng et al. 2026).

Traditionally, only three canonical species, *P. sinensis*, *P. rupestris*, and *P. filchnerae*, were recognized within sect. *Auganthus* (Hao et al. 2002). In recent years, with the advancement of floristic field investigations and the application of integrative taxonomic evidence, including morphological comparison, chloroplast genome- and nuclear SNP-based phylogeny, as well as reproductive isolation analyses, numerous new species have been successively published in this section. *P. xingshanensis* was described in 2023, followed by *P. rongrong*, *P. fujiangensis*, and *P. jiangyouensis* in 2025, and *P. maershanensis* was newly reported in 2026 (Wang et al. 2023; Gu et al. 2025; Zhang et al. 2025; Sheng et al. 2026). At present, sect. *Auganthus* comprises eight species endemic to China. These findings have greatly enriched the species diversity of the section and implied that its actual species richness is far beyond previous estimates.

Most species of sect. *Auganthus* are narrowly restricted to specialized microhabitats such as karst valleys and limestone or conglomerate cliff crevices, with remarkably fragmented and geographically isolated distributions. Moreover, widespread morphological convergence and frequent differentiation of heterostylous breeding systems have posed great challenges to the delimitation of closely related species, and the evolutionary patterns within the section remain insufficiently clarified (Zhang et al. 2025; Sheng et al. 2026). Meanwhile, most newly described species in this section possess extremely narrow distribution ranges and small population sizes. Threatened by tourism development, human disturbance, and climate change, most of them have been assessed as Critically Endangered (CR), highlighting an urgent need for taxonomic clarification and fundamental conservation research.

During a recent field investigation of wild plant resources in Pingwu County, Sichuan Province, a morphologically distinct *Primula* population was discovered. This population exhibits the typical sectional features of sect. *Auganthus*, including hairy vegetative parts and inflated calyx bases, but displays stable and distinguishable morphological differences from all known congeners of the section. Based on morphological observations of wild populations and preliminary verification of its phylogenetic placement, this population is confirmed as an undescribed new species. In this study, a detailed morphological description of the new species is provided, along with comparisons with morphologically allied taxa, a discussion of its phylogenetic position, and an evaluation of its conservation status. This work will provide new baseline data for the systematics and species differentiation mechanisms of the genus *Primula*.

## Materials and methods

### Morphological description and comparison

**Figure 1.**
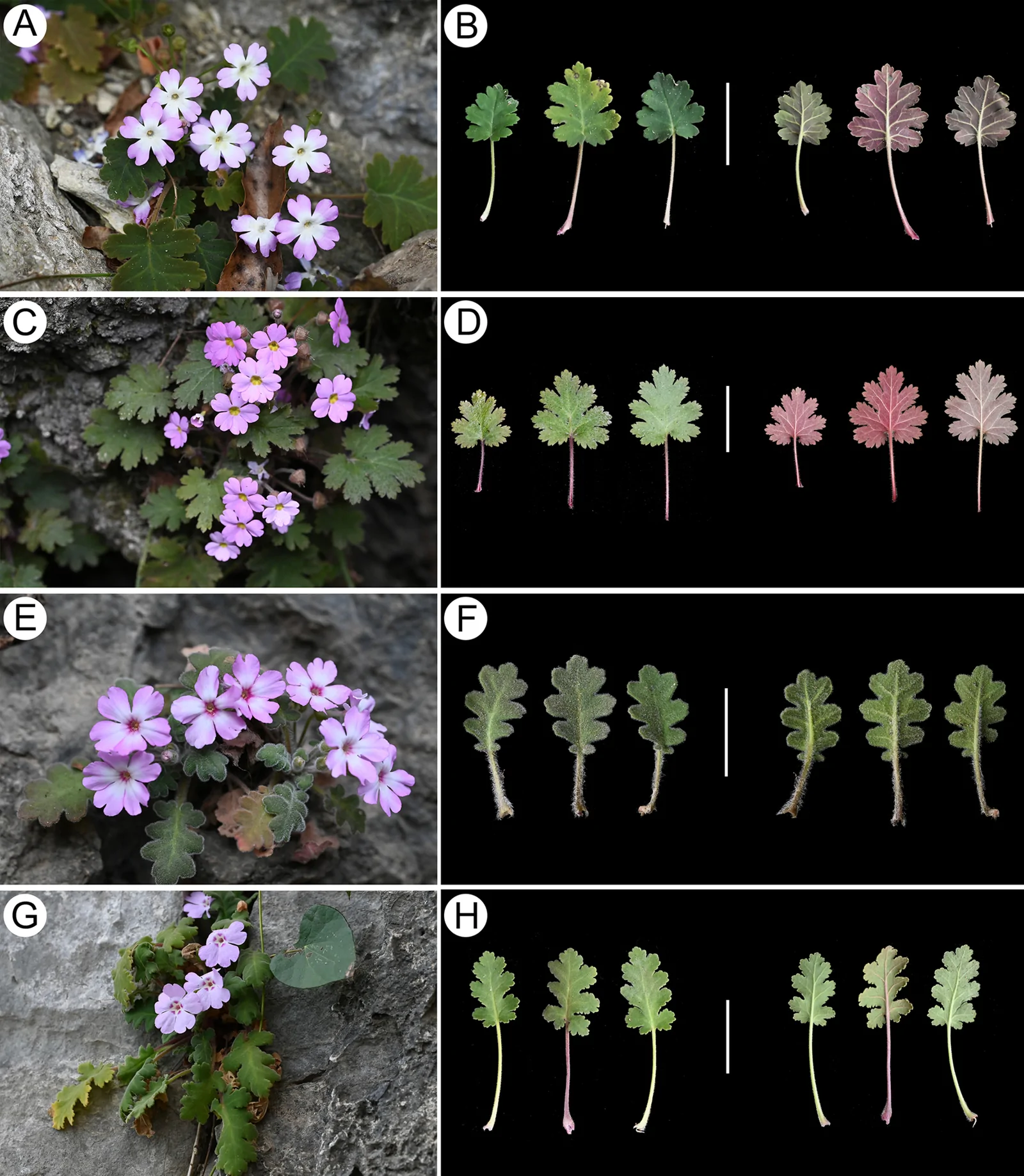
Morphological comparison between the new species and its close relatives. **A, B**. *Primula pingwuensis* sp. nov.; **C, D**. *P. sinensis*; **E, F**. *P. rongrong*; **G, H**. *P. fujiangensis*. The left column shows field photographs of the whole plant; the right column presents close-up images of leaves. The three leaves on the left side of the right column show the adaxial surfaces, and the three on the right show the abaxial surfaces. Scale bars: 5 cm.

Morphological observations, measurements, and descriptions of the new species were carried out based on living plants collected from its type locality in Pingwu County, Sichuan Province. The closely related species of *Primula* sect. *Auganthus*, including *P. sinensis*, *P. rongrong*, and *P. fujiangensis*, were selected for comparison (Fig. 1). These taxa are clustered in the same phylogenetic clade as the new species and are morphologically characterized by pink flowers and partially lobed leaves (Hu 1990; Hu and Kelso 1996; Zhang et al. 2025). Using population materials corresponding to the molecular phylogenetic sampling, 15 mature individuals were randomly selected from each population for the observation, measurement, and comparison of quantitative morphological traits (Sheng et al. 2026).

### Molecular phylogenetic analysis

**Figure 2.**
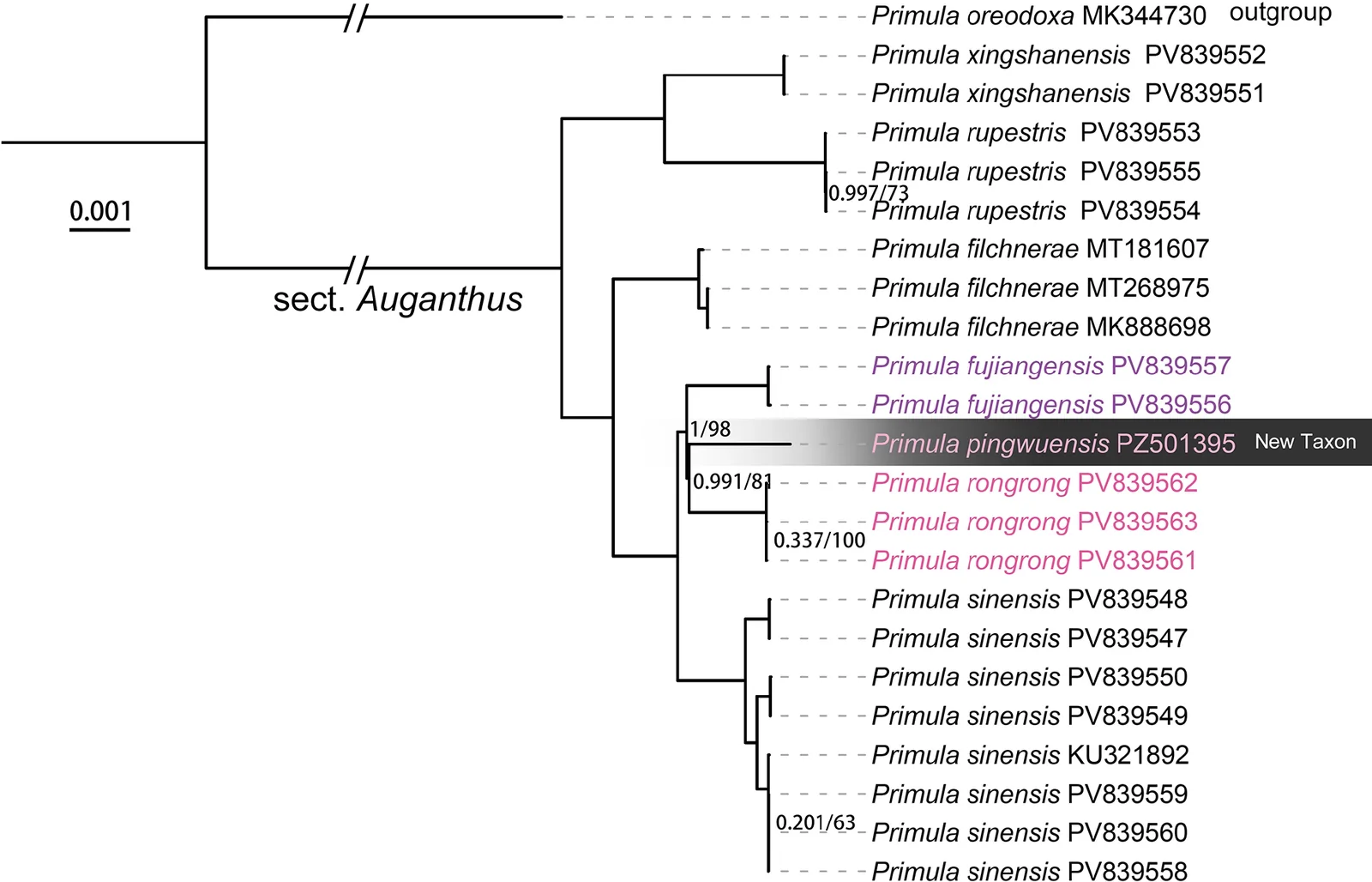
Phylogenetic tree of *Primula* sect. *Auganthus* based on chloroplast genomes from 23 individuals. Numbers above branches are BPP/BS values; branches without labeled support values indicate BPP = 1.0 and BS = 100.

**Figure 3.**
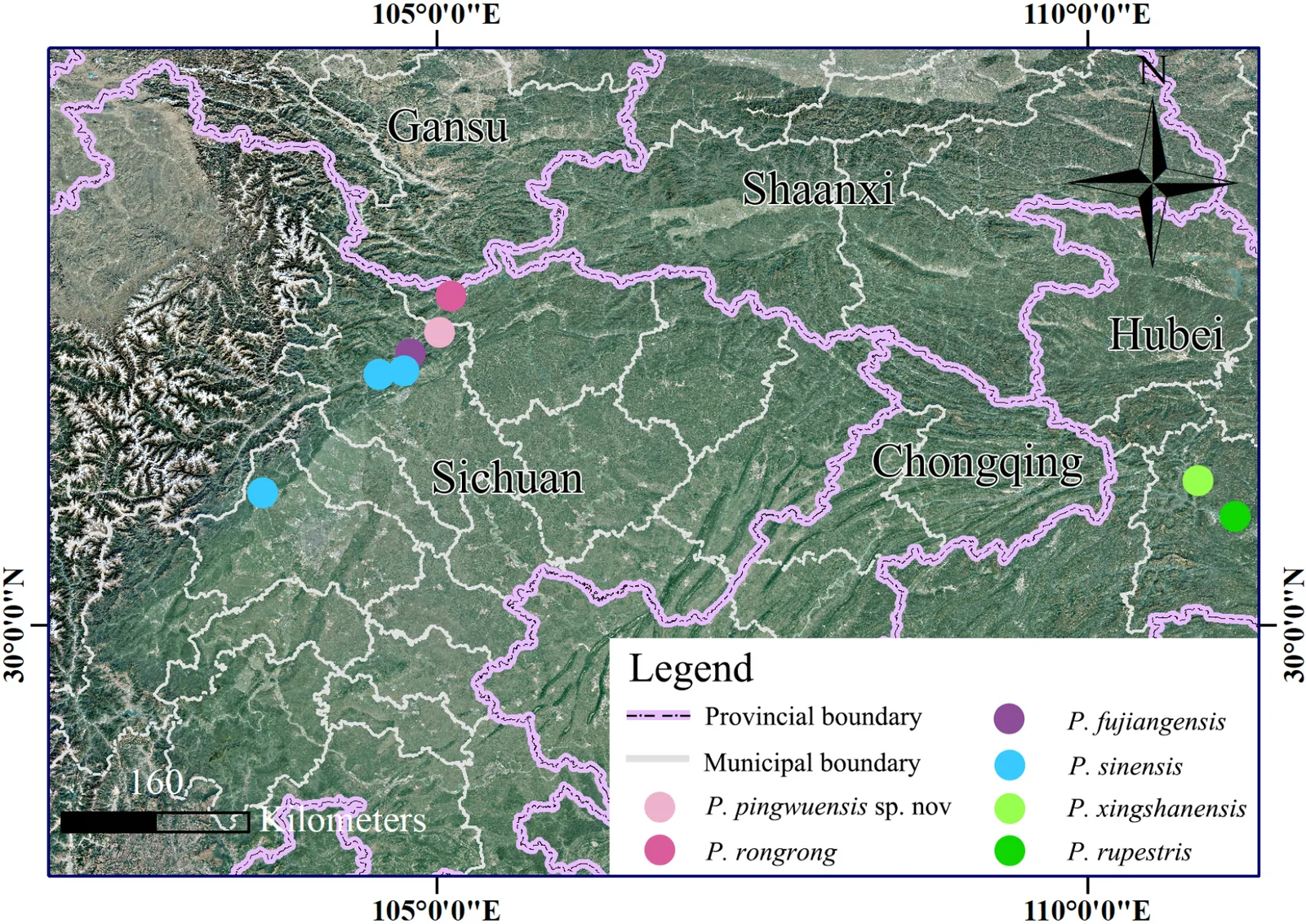
Georeferenced occurrence points with precise collection localities for phylogenetic analyses.

DNA was extracted from silica gel-dried leaf samples using the modified CTAB (mCTAB) method (Doyle and Doyle 1987; Larridon et al. 2015). The integrity and purity of the extracted DNA were assessed using a NanoDrop 1000 spectrophotometer and 0.8% agarose gel electrophoresis. Library construction and 150-bp paired-end sequencing were outsourced to Frasergen Biotechnology Co., Ltd. (Wuhan, China) on the Illumina HiSeq 6000 platform. For each sample, 1 μg of DNA was used for library preparation with the VAHTS Universal Plus DNA Library Prep Kit for Illumina. Dual 8-bp indexes (i7 + i5, TruSeq CD Set) were added, with a Hamming distance ≥ 3. Library quality control (QC) followed the criteria below: Qubit concentration ≥ 20 nM; the main fragment peak detected by a Bioanalyzer was 450 ± 50 bp; and adapter dimer content was less than 5%. Approximately 3 Gb of raw data were generated per sample for chloroplast genome assembly and annotation. The complete chloroplast genome was assembled using GetOrganelle v.1.7.1 and annotated via PGA (Qu et al. 2019; Jin et al. 2020). In this study, one newly sequenced chloroplast genome of the new species *Primula pingwuensis* was obtained. The accession numbers and relevant information for other *Primula* chloroplast genomes used herein are listed in Table 1 and Fig. 2. The distribution records of species with precise collection localities are shown in Fig. 3.

**Table 1.** Species and sequence information used for phylogenetic analyses.

**Locations**	**GenBank acc. no**
***Primula pingwuensis* sp. nov**.	
Jiangyouguan Town, Pingwu County, Mianyang City, Sichuan Province	PZ501395
***P. rongrong***	
Fangshi Town, Qingchuan County, Guangyuan City, Sichuan Province	PV839561, PV839562, PV839563
***P. fujiangensis***	
Yongsheng Town, Jiangyou City, Mianyang City, Sichuan Province	PV839556, PV839557
***P. sinensis***	
Longchi Town, Dujiangyan City, Chengdu City, Sichuan Province	PV839558, PV839559, PV839560
Hanzeng Town, Jiangyou City, Mianyang City, Sichuan Province	PV839549, PV839550
Qushan Town, Beichuan County, Mianyang City, Sichuan Province	PV839547, PV839548
Cultivated at South China Botanical Garden, Chinese Academy of Sciences, Guangzhou, Guangdong Province	KU321892
***P. filchnerae***	
Zhuxi County, Shiyan City, Hubei Province	MK888698
Zhuxi County, Shiyan City, Hubei Province	MT268975
Yangxian County, Hanzhong City, Shaanxi Province	MT181607
***P. rupestris***	
Xiaoxita Subdistrict, Yiling District, Yichang City, Hubei Province	PV839553, PV839554, PV839555
***P. xingshanensis***	
Xiakou Town, Xingshan County, Yichang City Hubei Province	PV839551, PV839552
***P. oreodoxa***	
Sichuan Province	MK344730

Phylogenetic trees were constructed based on the chloroplast genome sequence matrix generated from the above analyses. All sequences were aligned with MACSE v.2 (Ranwez et al. 2018). The optimal nucleotide substitution model was selected using ModelFinder (Minh et al. 2013; Kalyaanamoorthy et al. 2017). Phylogenetic relationships were reconstructed using maximum likelihood (ML) and Bayesian inference (BI) methods.

The ML analysis was performed in IQtree v.1.6.8 under the GTR+G+I model with 1000 bootstrap replicates. The Bayesian analysis was run in MrBayes v.3.2.6 with eight independent Markov chains for 50 million generations, with sampling every 1000 generations (Ronquist et al. 2012). All phylogenetic analyses were conducted using Phylosuite (Zhang et al. 2020).

## Result

### Morphological comparison

*Primula pingwuensis* is morphologically similar to *P. sinensis* in having palmately lobed leaves and multi-whorled inflorescences but can be readily distinguished from both *P. rongrong* and *P. fujiangensis*, which instead have pinnately lobed leaves and fewer flower whorls. Further comparison with *P. sinensis* reveals consistent differences in several key characters: *P. pingwuensis* possesses multi-branched stems, a densely pubescent indumentum, thickly chartaceous leaves that are elliptic-ovate to suborbicular in outline and 3–6 cm wide, with the abaxial surface ranging from pale green to purplish-red, and 2–3 whorls per scape with indistinct translucent nectar guides. In contrast, *P. sinensis* has few-branched stems, a densely hirsute indumentum, submembranous leaves that are ovate to subrotund and larger (4–12 cm wide), with the abaxial surface ranging from purplish-red to pale purple, and typically 1–3(5) whorls per scape with purple or yellow nectar guides. Detailed morphological comparisons between *P. pingwuensis* and its closely related species are presented in Table 2.

**Table 2.** Morphological comparison of *Primula pingwuensis* and its closely related species.

**Characters**	***P. pingwuensis***	***P. rongrong***	***P. fujiangensis***	***P. sinensis***
Stem	multi-branched	multi-branched	multi-branched	few-branched
Indumentum	densely pubescent	densely villous	densely pubescent	densely hirsute
Leaf texture	thickly chartaceous	thickly chartaceous, slightly fleshy	thickly chartaceous	submembranous
Leaf shape	elliptic-ovate to suborbicular	oblong	oblong	ovate to subrotund
Leaf width	3–6 cm	1–3 cm	1–3 cm	4–12 cm
Leaf lobing	palmately	pinnately	pinnately	palmately
Lobes	sparse coarse teeth	sparse rounded teeth	numerous coarse teeth	numerous coarse teeth
Leaf abaxial surface color	pale green to purplish-red	green	pale green, occasionally pale purple	purplish-red to pale purple
Number of whorls	2–3	1–2	1	1–3(5)
Flower color	externally pink, centrally nearly white	externally pink or light pink, internally white, centrally nearly white, light yellow or purple	externally pink or light pink, centrally white	externally purple, pink to white, centrally yellow
Nectar guides	indistinct translucent nectar guides	absent	purple	purple or yellow

### Molecular phylogenetic relationship

Phylogenetic trees reconstructed using maximum likelihood (ML) and Bayesian inference (BI) methods based on complete chloroplast genomes yielded identical topologies, which were consistent with the previously published phylogeny of *Primula* sect. *Auganthus* by Zhang et al. (2025). All interspecific branches received robust support (bootstrap support [BS] ≥ 70, Bayesian posterior probability [BPP] ≥ 0.95). Due to weak genetic differentiation within species, some intraspecific population nodes exhibited low Bayesian posterior probabilities but high bootstrap support values. Cladistic analyses revealed that the new species *P. pingwuensis* forms a sister group to *P. rongrong*, described in 2025; their common ancestral lineage is sister to *P. fujiangensis*. Furthermore, the new species exhibits a distinctly separate phylogenetic position from its morphologically closely related congener *P. sinensis*.

## Discussion

Phylogenetic analyses based on chloroplast genomes confirmed that *Primula pingwuensis* forms a sister lineage to *P. rongrong*, and the clade comprising these two species is sister to *P. fujiangensis*. Despite their close phylogenetic relationship, consistent morphological differences can be observed between *P. pingwuensis* and the latter two taxa, particularly in leaf lobing pattern, inflorescence architecture, and leaf texture. The consistent morphological divergence, combined with the clear genetic separation revealed by chloroplast data, provides morphological and plastid genomic evidence supporting the species status of *P. pingwuensis*. Based on plastid phylogeny and morphological divergence, this taxon appears to occupy a separate evolutionary branch distinct from its close relatives, with consistent morphological traits that readily distinguish it from allied species.

In *Primula* sect. *Auganthus*, palmate and pinnate leaf lobing do not have a single origin; multiple independent evolutionary transitions of this trait have been detected. The co-occurrence of palmately lobed *P. pingwuensis* and its pinnately lobed sister species indicates that leaf morphological traits can evolve rapidly even in closely related taxa. This trait variation is likely associated with morphological plasticity and adaptive differentiation. Diverse karst microhabitats may have driven the adaptive radiation and phenotypic differentiation observed within this clade, facilitating the formation of divergent morphological traits. The results also suggest that taxonomic frameworks relying on a small set of morphological characters, such as leaf lobing, cannot accurately reflect phylogenetic relationships in *Primula*. Accordingly, molecular phylogenetic evidence should be integrated for comprehensive taxonomic evaluation.

### Taxonomic treatment

#### Primula pingwuensis

Taxon classificationPlantaeEricalesPrimulaceae

Y.Hu, J.L.Gu & S.Y.Zhang sp. nov.

02B5D451-E499-5D10-A0DF-A686242E6A83

urn:lsid:ipni.org:names:77395660-1

Fig. 4

##### Type.

China • Sichuan, Mianyang City, Pingwu County, Jiangyouguan Town, 32°08'17.0"N, 104°47'27.0"E, under the deciduous broad-leaved forest on the hillside, 730 m a.s.l., 2 February 2026, *S.Y. Zhang, J.L. Gu & Y. Hu, ZSY20260201* (holotype: ANUB100131!; isotypes: ANUB100132!).

**Figure 4.**
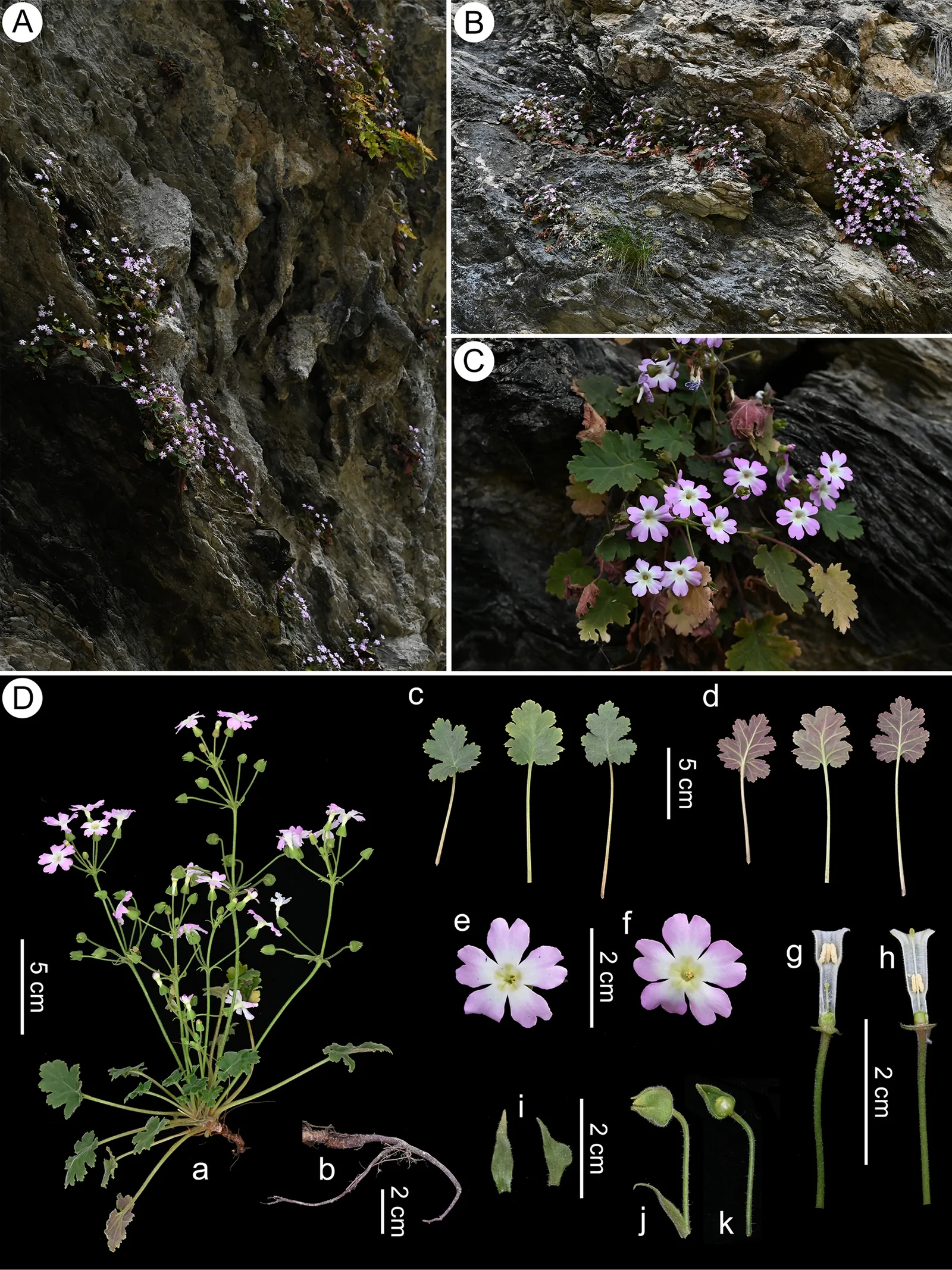
*Primula pingwuensis* sp. nov. **A, B**. Habitat; **C**. Flowering plant in the wild; **Da**. Flowering plant; **Db**. Root; **Dc**. Adaxial surface; **Dd**. Abaxial surface; **De**. Front view of pin flower; **Df**. Front view of thrum flower; **Dg**. Longitudinal section of thrum flower; **Dh**. Longitudinal section of pin flower; **Di**. Bract; **Dj**. Young fruits; **Dk**. Young fruit, showing ovary.

##### Diagnosis.

*Primula pingwuensis* is morphologically similar to *P. sinensis* in having palmately lobed leaves and multi-whorled scapes but differs by its multi-branched stems, densely pubescent indumentum, thickly chartaceous leaves (vs. submembranous in *P. sinensis*), narrower blades (3–6 cm wide), 2–3 flower whorls, and indistinct translucent nectar guides. *Primula pingwuensis* shares a close evolutionary affinity with *P. rongrong* and *P. fujiangensis*. The three taxa exhibit high morphological resemblance and overlapping geographical ranges. *P. pingwuensis* bears palmately lobed foliage, with all vegetative and floral parts shortly and densely pubescent, plus faint inconspicuous nectar guides. In contrast, both allied species produce pinnately divided leaves: *P. rongrong* is fully villous and lacks nectar channels, whereas *P. fujiangensis* is densely pubescent and bears conspicuous purple nectar ducts. This set of consistent morphological characters readily distinguishes *P. pingwuensis* from the two allied taxa.

##### Description.

Perennial herb, 8–20 cm tall. Rhizomes brown, 3–10 cm long, branched near the apex, comparatively stout. Leaves 7–15 in an open rosette; petioles 5–15 cm long, green or yellowish-brown, broadly flattened at the base, densely white-pubescent; leaf blades elliptic-ovate to suborbicular, 4–8 × 3–6 cm, chartaceous-fleshy, shortly and densely white-pubescent; adaxially green to yellow-green, abaxially pale green to purplish-red; palmately lobed to 1/3–1/2 of its width, lobes usually 7, rectangular, with 3–5 fine serrations along the margin; base cordate, lateral veins 3–4 on each side. Scape solitary, arising from each leaf rosette, 10–20 cm tall, densely white-pubescent; umbels 2–3 whorled, each whorl with 3–7 flowers; bracts lanceolate to long-triangular, 8–15 mm × 2–8 mm, apex acute, densely white-pubescent; pedicels 2.0–3.5 cm long, densely white-pubescent. Glandular hairs absent on pedicels and calyx. Flowers heterostylous; calyx bell-shaped, 5–7 mm long, 5–7 mm in diam., inflated at base in fruit, lobed to approximately 1/2 of its length, lobes triangular, margin entire, densely white-pubescent; corolla pink, puberulous on both surfaces; throat white, with indistinct pale yellow or translucent nectar guides; limb 2.0–2.6 cm in diam., lobes 5, obovate, 2-lobed at apex, parted to 1/6–1/4 of the lobe length. Pin flowers with corolla tube ca. 10 mm long, stamens attached below the middle, style reaching or slightly protruding from the corolla throat, 10–12 mm long; thrum flowers with corolla tube ca. 10 mm long, stamens inserted near the corolla throat, style 5–7 mm long. Stigma globular; ovary globular, glabrous, ca. 2 mm in diam., ovules numerous. Capsule subglobose, 6–8 mm in diam., dehiscing at the apex when mature, usually with seven triangular lobes. Seeds numerous, black, ellipsoid, approximately 1 mm long.

##### Phenology.

Flowering from January to March, fruiting from May to June.

##### Distribution and habitat.

It grows on shaded conglomerate cliff faces in valleys at altitudes of 600–800 m. At present, it is known only from Pingwu County, where two populations occur approximately 30 km apart.

##### Chinese name.

Píng Wŭ Bào Chūn (平武报春).

##### Etymology.

The specific epithet is derived from Pingwu County, Mianyang City, Sichuan Province, the type locality of the new species, which is also an important habitat of the giant panda.

##### Provisional conservation status.

*Primula pingwuensis* is currently known from only two localities, with an area of occupancy (AOO) of less than 10 km^2^. Intensive human activities take place around both distribution sites, although no obvious negative impacts on wild populations have yet been documented. However, long-term population and habitat monitoring data are absent, so it cannot be confirmed whether its populations or habitats are undergoing continuous decline. Given the insufficient ecological and demographic data available for formal threatened-category evaluation, this species is assessed as Data Deficient (DD) under the IUCN Red List Categories and Criteria (Version 16.0, IUCN 2024).

### Key to the species of *Primula* sect. *Auganthus*

**Table:** 

1	Flowers yellow	**2**
–	Flowers white, pale lilac, pink, or rose	**3**
2	Flowers homostylous; leaf blade suborbicular, slightly wider than long, margin palmately lobed	***P. maershanensis***
–	Flowers heterostylous; leaf blade oblong-ovate to ovate-orbicular, longer than wide, margin pinnately lobed	***P. jiangyouensis***
3	Leaf blades oblong, length far exceeding width	**4**
–	Leaf blades ovate to subrounded, slightly longer than broad	**7**
4	Leaves pinnately lobed, lobing extending to the midvein	***P. filchnerae***
–	Leaves pinnately or palmately lobed, lobing not reaching the midvein	**5**
5	Leaf base cuneate; lobes entire	***P. xingshanensis***
–	Leaf base cordate; lobes serrate	**6**
6	Whole plant villous; nectary ducts absent	***P. rongrong***
–	Whole plant hairy; nectary ducts purple	***P. fujiangensis***
7	Leaf rosette base bearing wiry persistent petioles of withered old leaves; fruiting calyx 8–10 mm long	***P. rupestris***
–	No persistent withered leaf stalks at the base of leaf rosettes	**8**
8	Calyx 15–20 mm in fruit; leaf blade submembranous	***P. sinensis***
–	Calyx 5–7 mm in fruit; leaf blade thickly chartaceous	***P. pingwuensis* sp. nov**.

## Supplementary Material

XML Treatment for Primula pingwuensis

## Additional information

### Artificial Intelligence (AI) use

The authors accept full responsibility for the content of the manuscript, including the disclosure of any use of AI.

No AI tools were used in the preparation of this manuscript.

### Author ORCIDs

S.-Y. Zhang https://orcid.org/0000-0003-2674-7340

M. Fang https://orcid.org/0009-0003-6491-4960

Y.-F. Hu https://orcid.org/0000-0002-1549-4387

Y. Hu https://orcid.org/0009-0003-4997-8317

J.-L. Gu https://orcid.org/0009-0007-7557-0445

Z.-K. Wu https://orcid.org/0000-0002-4583-7782

J.-W. Shao https://orcid.org/0000-0002-4816-8457

### Data availability

All of the data that support the findings of this study are available in the main text.

## References

[B1] Balfour IB (1913) Chinese species of *Primula*. Journal of the Royal Horticultural Society of London 39: 128–167.

[B2] Balfour IB, Farrer RJ (1918) Some new species of *Primula* which have flowered recently. Transactions of the Botanical Society of Edinburgh 27(3): 240–243. 10.1080/03746601809468482

[B3] Doyle JJ, Doyle JL (1987) A rapid DNA isolation procedure for small quantities of fresh leaf tissue. Phytochemical Bulletin 19: 11–15.

[B4] Gilmartin PM (2015) On the origins of observations of heterostyly in *Primula*. New Phytologist 208: 39–51. 10.1111/nph.13558 26255981

[B5] Gu JL, Wang HD, Lin HQ, Jiang XQ, Shuai T, Wu ZK (2025) *Primula jiangyouensis* (Primulaceae), a new species of *Primula* sect. *Auganthus* from Sichuan, China. PhytoKeys 260: 1–25. 10.3897/phytokeys.260.158039 PMC12284580 40704085

[B6] Hao G, Hu QM, Lee MS (2002) Circumscriptions and phylogenetic relationships of *Primula* sects. *Auganthus* and *Ranunculoides*: Evidence from nrDNA ITS sequences. Acta Botanica Sinica 44(1): 72–75.

[B7] He X, Cao JJ, Zhang W, Li YQ, Zhang C, Li XH, Xia GH, Shao JW (2021) Integrative taxonomy of herbaceous plants with narrow fragmented distributions: A case study on *Primula merrilliana* species complex. Journal of Systematics and Evolution 60(4): 859–875. 10.1111/jse.12726

[B8] Hu CM (1990) Primulaceae. In: Chen FH, Hu CzM (Eds) Flora Republicae Popularis Sinicae. Science Press, Beijing 59, 277 pp.

[B9] Hu CM, Kelso S (1996) Primulaceae. In: Wu ZY, Raven PH (Eds) Flora of China. Science Press, Beijing & Missouri Botanical Garden Press, St. Louis 15, 99–185.

[B10] IUCN [Standards and Petitions Committee] (2024) Guidelines for Using the IUCN Red List Categories and Criteria. Version 16. Prepared by the Standards and Petitions Committee. https://www.iucnredlist.org/documents/RedListGuidelines.pdf [Accessed on 25 May 2026]

[B11] Jin JJ, Yu WB, Yang JB, Song Y, Claude W, Yi TS, Li DZ (2020) GetOrganelle: A fast and versatile toolkit for accurate *de novo* assembly of organelle genomes. Genome Biology 21: 241. 10.1186/s13059-020-02154-5 PMC7488116 32912315

[B12] Kalyaanamoorthy S, Minh BQ, Wong TKF, von Haeseleret A, Jermiin LS (2017) ModelFinder: fast model selection for accurate phylogenetic estimates. Nature Methods 14: 587–589. 10.1038/nmeth.4285 PMC5453245 28481363

[B13] Larridon I, Walter HE, Guerrero PC, Duarte M, Cisternas MA, Hernández CP, Bauters K, Asselman P, Goetghebeur P, Samain M (2015) An integrative approach to understanding the evolution and diversity of *Copiapoa* (Cactaceae), a threatened endemic Chilean genus from the Atacama Desert. American Journal of Botany 102(9): 1506–1520. 10.3732/ajb.1500168 26373974

[B14] Ma YP, Tian XL, Zhang JL, Wu ZK, Sun WB (2014) Evidence for natural hybridization between *Primula beesiana* and *P. bulleyana*, two heterostylous primroses in NW Yunnan, China. Journal of Systematics and Evolution 52: 500–507. 10.1111/jse.12077

[B15] Minh BQ, Nguyen MA, Haeseler A (2013) Ultrafast approximation for phylogenetic bootstrap. Molecular Biology and Evolution 30(5): 1188–1195. 10.1093/molbev/mst024 PMC3670741 23418397

[B16] POWO (2026) Plants of the World Online. Facilitated by the Royal Botanic Gardens, Kew. http://www.plantsoftheworldonline.org/ [Accessed on 25 May 2026]

[B17] Qu XJ, Moore MJ, Li DZ, Yi TS (2019) PGA: a software package for rapid, accurate, and flexible batch annotation of plastomes. Plant Methods 15: 50. 10.1186/s13007-019-0435-7 PMC6528300 31139240

[B18] Ranwez V, Douzery EJP, Cambon C, Chantret N, Delsuc F (2018) MACSE v2: Toolkit for the alignment of coding sequences accounting for frameshifts and stop codons. Molecular Biology and Evolution 35: 2582–2584. 10.1093/molbev/msy159 PMC6188553 30165589

[B19] Richards J (2002) *Primula* (2^nd^ edn). Batsford, London, UK, 29–30.

[B20] Ronquist F, Teslenko M, van der Mark P, Ayres DL, Darling A, Höhna S, Larget B, Liu L, Suchard MA, Huelsenbeck JP (2012) MrBayes 3.2: efficient Bayesian phylogenetic inference and model choice across a large model space. Systematic Biology 61: 539–542. 10.1093/sysbio/sys029 PMC3329765 22357727

[B21] Sheng MY, Wu YM, Gu JL, Lin HQ, Zhou W, Zheng L, Wu ZK (2026) *Primula maershanensis* (Primulaceae), a new species in *Primula* sect. *Auganthus* from Sichuan, China. PhytoKeys 274: 61–71. 10.3897/phytokeys.274.191386 PMC13146781 42099569

[B22] Wang SQ, Yan HF, Cheng ZJ, Wang YB (2023) *Primula xingshanensis* (Primulaceae), a new species from Hubei, China. Phytotaxa 594(2): 158–162. 10.11646/phytotaxa.594.2.8

[B23] Zhang D, Gao FL, Jakovlić I, Zou H, Zhang J, Li WX, Wang GT (2020) PhyloSuite: An integrated and scalable desktop platform for streamlined molecular sequence data management and evolutionary phylogenetics studies. Molecular Ecology Resources 20(1): 348–355. 10.1111/1755-0998.13096 31599058

[B24] Zhang SY, Hu YF, Gu JL, Hu Y, Si XY, Zhang W, Shao JW (2025) Two new species of *Primula* sect. *Auganthus* from Sichuan, China. Ecology and Evolution 15(9): e72047. 10.1002/ece3.72047 PMC12391589 40896101

[B25] Zhang W, Hu YF, Zhang SY, Shao JW (2023) Integrative taxonomy in a rapid speciation group associated with mating system transition: A case study in the *Primula cicutariifolia* complex. Molecular Phylogenetics and Evolution 186: e107840. 10.1016/j.ympev.2023.107840 37279815

